# Characterization of non-tuberculous mycobacterium from humans and water in an Agropastoral area in Zambia

**DOI:** 10.1186/s12879-017-2939-y

**Published:** 2018-01-08

**Authors:** Ngula Monde, Musso Munyeme, Adrian Muwonge, John Bwalya Muma, Sydney Malama

**Affiliations:** 1 Department of Biomedical Sciences, Tropical Diseases Research Center, P.O.Box 71769, Ndola, Zambia; 2 Department of Disease Control, School of Veterinary Medicine, University of Zambia, P.O. Box 32379, 10101 Lusaka, Zambia; 3 University of Edinburgh, Roslin Institute, Easter Bush, Midlothian, Scotland EH259RG UK; 4 Health Promotions Unit, Institute of Economic and Social Research, University of Zambia, P.O. Box 30900, Lusaka, Zambia

**Keywords:** Namwala, NTM, Water, Mycobacteria, Zambia

## Abstract

**Background:**

The non-tuberculous mycobacteria include those mycobacterium species that are not members of the Mycobacterium tuberculosis complex, the causative agent of pulmonary tuberculosis and *Mycobacterium leprae*. In Zambia, Non-tuberculous Mycobacteria are gaining recognition as pathogens of public health significance. However, there is scanty information on the isolation and speciation of these organisms for better patient management, consequently reducing the burden of these infections. Given the above information, the thrust of this study was to isolate and characterize NTM from humans and water in Namwala district of Zambia.

**Method:**

This was a cross-sectional study were 153 individuals with suspected TB were sampled from four health facilities in Namwala district, sputum samples were also collected. Additionally, 149 water samples were collected from different water drinking sources such as Tap water, Borehole water, rivers, wells and streams. Standard TB culture methods were employed to isolate Non-tuberculous Mycobacteria and later *16S–23S* internal transcribed spacer region Sequencing was employed to characterize NTM.

**Results:**

Seven (7, 4.6%) NTM species were identified from humans with *M. arupense* (3, 42.9%) being the most common organism, while twenty three (23, 15.4%) NTM were identified from water with the common species being *Mycobacterium gordonae (*5, 21.7%). *Mycobacterium avium* and *Mycobacterium fortuitum* were both identified from human and water samples*.*

**Conclusion:**

This study has shown the isolation of NTM species from humans and water. The isolation of NTM from drinking water sources could signify a public health risk to humans.

## Background

The Non-tuberculous Mycobacteria (NTM) include those mycobacterium species that are not members of the Mycobacterium tuberculosis complex (MTC), the causative agent of pulmonary tuberculosis (TB) and *Mycobacterium laprae* [2, 3, 24]. They are normal inhabitants of natural waters, drinking waters, animals, birds and soils [25, 33, 41]. The environment is the primary source of infection of these mycobacteria to humans. NTM have also been recovered from meat, fish, dairy products, fruits, vegetables and specially unpasteurized milk suggesting a public health risk for these bacteria [19].

Human-to-human transmission of NTM is generally uncommon, although there is evidence of transmission of certain NTM species such as *Mycobacterium kansasii* [33].

Although members of the MTC are largely known to cause the majority of the mycobacterial infections worldwide, infections due to NTM are also emerging [17]. There are 172 different species of NTM but the most potentially pathogenic of these are *Mycobacterium avium, Mycobacterium intracellulare, Mycobacterium kansasii, Mycobacterium xenopi, and Mycobacterium abscessus* [17]. These organisms are capable of causing pulmonary disease, disseminated disease and localized lesions, mostly in immune-compromised individuals. Pulmonary diseases are the most frequently encountered and have been reported to account for up to 94% of cases of NTM associated disease [28]. People living with Human Immunodeficiency Virus (HIV) and Acquired Immunodeficiency Syndrome (HIV/AIDS), represent one of the most vulnerable populations to NTM infections, as well as a higher risk for complications and poor disease outcomes. Those receiving immunosuppressive therapy secondary to organ transplantation, cancer, autoimmune disease and those with diabetes mellitus may also be more susceptible to NTM infections compared to the general population [2].

Non-tuberculous Mycobacteria can display clinical and radiological features similar to those exhibited by MTC, hence the need for species identification. Distinguishing between MTC and NTM infections may be challenging, especially in low income countries such as Zambia where Acid Fast Bacilli (AFB) smear microscopy is mainly used for MTC diagnosis. Even though AFB smear microscopy, allows for rapid diagnosis of mycobacteria, it does not differentiate MTC from NTM. This may lead to NTM related infections being under-diagnosed or misdiagnosed as TB [7].

In Zambia, NTM have also been recognized as pathogens of major public health significance with the advent of HIV and AIDS [5, 6, 21]. However, only scanty studies have demonstrated the isolation and characterization of mycobacteria species in humans and water. This study therefore, aimed at isolation and characterization of NTM from humans and water in Namwala district of Zambia.

## Methods

### Study area

**Fig. 1:**
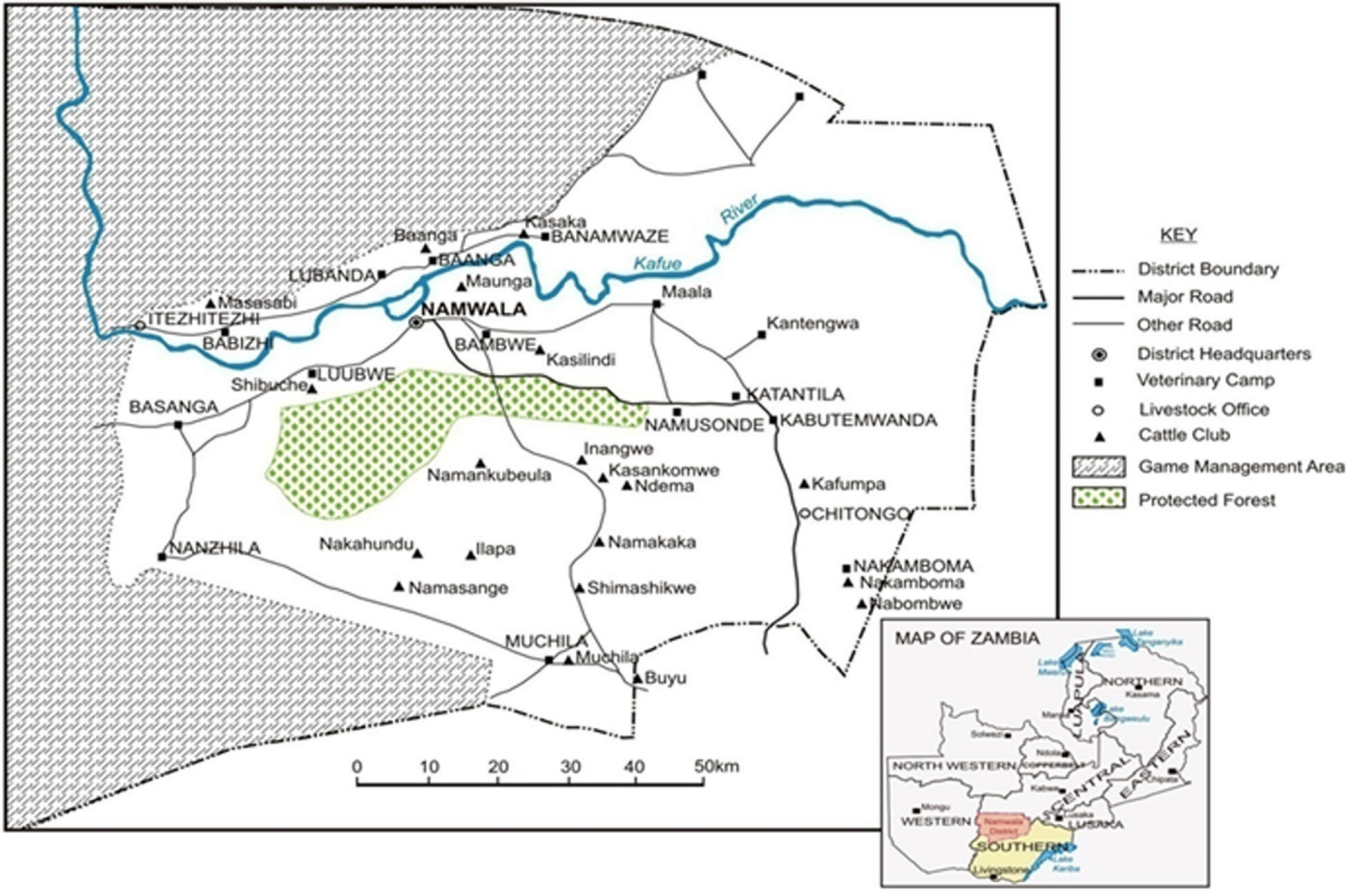
Map of Namwala district

The study was conducted in Namwala district of Zambia. Namwala is a rural district situated in the southern province of Zambia and is one of the districts with a large number of pastoral farmers. It has the highest TB prevalence in both animals and humans [22, 23]. It covers an estimated total area of about 10,000 km^2^ and lies between latitudes 15 and 17°S of the equator and longitude 25 and 27° E. (Fig. 1). The greater area of its traditional land is covered by the Kafue flood plains of the Kafue River. These offer nutritive varieties of rich lush green grass for both cattle and wildlife for a greater part of the year than the surrounding Savannah woodlands [24]. It supports approximately 30,000 herds of cattle and 44,000 Kafue lechwe [22]. Agriculture is the main economic activity, and majority of the people keep livestock. Humans and animals commonly share the same micro-environments such as water drinking points, especially in dry seasons [30], increasing the possibility of interspecies disease transmission or contamination of water sources, which could potentially lead to a zoonotic transmission to humans [18].

### Study design

This was a cross-sectional study in adolescent and adult presumptive TB patients. In addition, water samples were collected. For human sampling, sputum samples were collected from patients seeking medical attention at four local health centers in Namwala district, namely, Namwala district Hospital, Chitongo Health Centre, Kabulamwanda Health Centre and Maala Rural Health Centre. Patients who presented with TB-like symptoms such as chronic (Defined as ≥ 2 weeks) productive cough, loss of appetite, fever, fatigue, headache and night sweats were enrolled in the study. Participants who presented with other ailment were not included in our study.

Water samples were collected from common water drinking points in areas where the study participants came from. The water collection points comprised of taps, communal boreholes, open wells, and streams/river. Community health workers in these areas helped in accessing these water points. Our sampling was purposive as study subjects were selected based on the above mentioned characteristics.

### Study period

The study was carried out between October 2015 and March 2016.

### Laboratory methods

#### Sputum samples

After routine microscopy with the Ziehl–Neelsen (ZN) staining technique, the samples were stored in cetylpyridinium chloride (CPC) transport medium (Sigma-Aldrich, Steinheim, Germany, a mixture of 1.0% CPC and 2.0%NaCl) and kept at ambient temperature at the health facilities until they were taken to Tropical Diseases Research Centre (TDRC), TB Reference Laboratory (Ndola) for processing. At TDRC TB Reference Laboratory, samples were decontaminated using the Petroff method [29] and cultured on Lowenstein-Jensen (LJ) medium. The cultures were incubated at 37 °C and examined for growth weekly for at least 8 weeks and results recorded. Microscopic examination of positive cultures using ZN staining method was performed to detect the presence of AFB.

#### Water samples

One hundred and forty nine (149) water samples were collected from various drinking points such as streams, boreholes, tap and rivers in Namwala Central, Maala, Kabulamwanda and Chitongo. An amount of 150 ml water was collected in sterile whirlpak plastic bags and immediately placed in a cool box containing ice packs and transported to the laboratory where they were stored in CPC media (BD Diagnostics, Sparks, MD) until processed.

Hundred and Fifty (150 ml) of water was filtered through 0.45 μm nitrocellulose membrane filters (Millipore Corporation, Bedford, MS, USA) by vacuum filtration using a Manifold Filtration System (Sartorius AG, Goettingen, Germany). The membranes were transferred into sterile screw-capped specimen containers with 10 ml of 0.005% CPC. The surface of the membranes was abraded vigorously and thoroughly with a sterile plastic inoculating loop. The samples were then exposed to 0.005% CPC for 30 min at room temperature. The mixture was then centrifuged at 3000×g for 20 min. The pellet was washed twice with 1 ml sterile normal saline and finally re-suspended in 1 ml sterile distilled water. The suspension (0.5 ml) was inoculated on LJ slants and incubated at 37 °C. The cultures were examined for growth for up to 8 weeks and results recorded. Microscopic examination of positive cultures was done using Ziehl Neelsen staining method to detect the presence of AFB.

#### Sequencing of 16S- 23S rRNA ITS region

Upon detection of AFB in positive cultures, the Mycobacterial growth was identified as either MTC or NTM using a rapid identification test known as Capilia TB-Neo Kit (TAUNS Laboratories, Inc. Japan), following manufacturer’s instructions (capilia.co.jp/english/capilia_tb-neo.html). To obtain genomic DNA for sequencing, the invitrogen TRIzol™ Reagent was used for DNA extraction according to Hongbao et al. [15]. Thereafter, polymerase chain reactions (PCRs) were performed followed by sequencing of the 16S – 23SrRNA Internal Transcribed Spacer region with primers Sp1 (5′-ACC TCC TTT CTA AGG AGC ACC-3′) and Sp2 (5′-GAT GCT CGC AAC CAC TAT CCA-3′) [34]. Sequencing was performed using an ABI sequencer (Applied Biosystem Inc.) at the school of Veterinary Medicine, University of Zambia. The obtained sequences were aligned using MEGA version 7and the alignments were then blasted against the NBCI database with Mycobacterium taxid 85,007 as the reference organism. The strain name with the highest E.value and identity hit and score was chosen.

## Results

### Isolation of NTM in sputum and water samples

Based on morphological characteristics on LJ media and acid fastness by the ZN method, 17 (11.11%) mycobacterial species were detected out of the 153 study participants. Seven (7) (4.6%) were identified as NTM while 10 (6.5%) were MTC. However, co-infection of NTM and MTC was not observed among the suspected TB patients.

Thirty two 32 (21.5%) NTM were isolated from 149 water samples collected. The majority of NTM were isolated from borehole water sources 17(53.1%) followed by river 9 (28.1%), stream 3 (9.4%), dam 2 (6.3%), and tap 1 (3.1%) water sources.

### Molecular characterization of NTM in humans and water

All the seven (7) NTM isolated were characterized to species level, with the most isolated species being *Mycobacterium arupense* 3 species (42.9%) (Table 1).

**Table 1:** NTM species isolated in Humans and Water in Namwala district

NTM species	*n*	Frequency (%)
Humans (*N* = 153, *n* = 7)		
*M. arupense*	3	42.9
*M. abscesus*	1	14.3
*M. avium*	1	14.3
*M. fortuitum*	1	14.3
*M. nebraskense*	1	14.3
Water (*N* = 149, *n* = 23)		
*M. gordonae*	5	21.7
*M. senegalense*	3	13.0
*M, fortuitum*	3	13.0
*M. peregrinum*	3	13.0
*M. kumamotonense*	1	4.3
*M. simiae*	1	4.3
*M. avium*	1	4.3
*M. gilvum*	2	8.7
*M. flavescens*	1	4.3
*M. housetonense*	1	4.3
*M. smegmatis*	1	4.3
*M. parafinicum*	1	4.3

Out of 32 NTM isolated from water samples, 23 NTM species were characterized with the most common being *Mycobacterium gordonae,* 5 species (21.7%); *Mycobacterium senegalense,* 3 species (13.0%); *Mycobacterium peregrinum,* 3 species (13.0%)*, Mycobacterium fortuitum,* 3 species (13.0%) and *Mycobacterium gilvum*, 2 species (8.7%) (Table 1). Nine (9) NTM were uncharacterized. *Mycobacterium avium and Mycobacterium fortuitum* were both isolated in humans and water samples. Of the four study sites in Namwala district, Maala had the most NTM species identified (11 species) while Chitongo had the least NTM species (2 species).

## Discussion

Non-tuberculous Mycobacteria have gained much clinical significance in the last couple of decades in both immuno-compromised and immune-competent individuals [1]. Water is a documented source of NTM infection in humans, but it is not the only source [38]. Currently, very little data exists on the NTM species distribution in humans and water in Africa, Zambia inclusive. It was against this background that this present study was formulated and to the best of our knowledge, this might be the first study in Zambia to isolate and characterize NTM from humans and water simultaneously.

In the present study, a range of NTM from humans and water in Namwala district, an agro-pastoral district located in Southern province of Zambia were isolated. The study found the overall prevalence of NTM in humans in Namwala district to be 4.6%, among patients presenting with TB-like symptoms at local health centers. This is comparable to a study done in Shanghai China, were the overall prevalence of NTM in humans was 5.9% [44].

In the current study, out of 149 water samples collected from various study sites in Namwala district, 32 (21.5%) samples were positive for NTM using phenotypic identification method, indicating that NTM were common in water sources of Namwala district.

Our study also found a high isolation rate of NTM in borehole water sources followed by river, dam, stream and lowest in tap water sources. The most prevalent NTM species isolated was *Mycobacterium gordonae*. Although another study carried out in Sri Lanka [10] showed a high frequency of isolation of mycobacteria from aquarium water and surface water (rivers, streams) with the most common NTM being *Mycobacterium fortuitum*), These findings in our study could be due to high levels of organic matter and soil in borehole waters and surface waters (rivers, dams, streams) contributing to the mycobacterial flora. The piping systems used in boreholes may support the formation of biofilms which favors the growth and multiplication of NTM [37]. Some pipe materials have been shown to contribute to biofilm formation particularly iron pipes [37] The capacity of mycobacteria to form biofilms has been demonstrated in studies done elsewhere [9, 13]. In Germany, 90% of biofilm samples from pipes of various water distribution systems contained Mycobacteria, signifying that Mycobacteria biofilms are present in piping systems [35]. The low mycobacterial load in tap water sources observed in our study could probably be related to the lethal effect of chlorine to mycobacteria, since tap water is usually chlorinated in Namwala district by the local water supply company.

The isolation rate of NTM in both water and human samples was also highest in Maala area as compared to the other study sites in Namwala district of Zambia. This can be attributed to the fact that Maala is surrounded by open grasslands with a lot of Dambos, rivers and papyrus flora, with reeds that enhance the formation of microfilms and subsequently increase the multiplication of NTM [13].

A range of pathogenic NTM species were also isolated in the present study and the most common organism was *Mycobacterium arupense*. This is the first study in Zambia to report the isolation of *Mycobacterium arupense* in human sputum samples. Cloud et al., [8] identified *Mycobacterium arupense* for the first time in human clinical samples in the United States of America. Other studies done in Japan [26], Taiwan [40]; and in Greece [27] also reported the isolation of *Mycobacterium arupense* in human sputum samples. In all these four studies *Mycobacterium arupense* was of clinical significance. *Mycobacterium arupense* is a novel mycobacterium species potentially pathogenic and has been associated with causing human pulmonary infections [36]. Therefore, *Mycobacterium arupense* isolated from humans in this study could possibly be of clinical significance as the patients presented with symptoms of pulmonary infection.

Other NTM species that were isolated in humans were *Mycobacterium abscessus*, *Mycobacterium avium*, *Mycobacterium fortuitum* and *Mycobacterium nebraskense*. All these species have been found to cause disease in immune compromised and immune competent individuals. *Mycobacterium abscessus* has been reported to be the leading cause of lower respiratory tract infections among the rapidly growing NTM species [6, 16]. *Mycobacterium avium* has also been known to cause opportunistic infections in humans and animals [11] while *Mycobacterium nebraskense* is an NTM pathogen that has been associated with nodular pulmonary disease [32].

Drinking water has been suggested as a reservoir for many pathogenic and potentially pathogenic NTM such as *Mycobacterium avium complex, Mycobacterium gordonae, Mycobacterium kansasii, Mycobacterium fortuitum, Mycobacterium simiae, Mycobacterium senegalense, Mycobacterium chelonae* and *Mycobacterium xenopi* [12, 31, 38]. In our study, a number of pathogenic and potentially pathogenic NTM species were isolated from water samples of Namwala district. The most prevalent NTM species was *Mycobacterium gordonae*, followed by *Mycobacterium fortuitum*, *Mycobacterium senegalense*, and *Mycobacterium peregrinum*. This is in partial agreement with the findings of the study conducted by Thomson and others in Brisbane, Australia, where *Mycobacterium gordonae* was the most commonly isolated species in natural and municipality water sources [38]. *Mycobacterium gordonae* is one of the least pathogenic of the mycobacterium species isolated. It is usually a contaminant or colonizer in immune competent individuals; however, it has been found to be pathogenic in HIV infected patients who are severely immune compromised [4]. Apart from AIDS, underlying immunue suppression, advanced structural lung disease such as emphysema, pneumoconiosis, alcoholism, chronic lung disease, diabetes mellitus and malignancy have all been reported as risk factors for invasive infections [4, 43]. Therefore, *Mycobacterium gordonae* isolated from clinical samples should not be automatically considered as a contaminant or colonizer especially in immune compromised patients or patients with the above mentioned risk factors.

*Mycobacterium fortuitum, Mycobacterium senegalense* and *Mycobacterium peregrinum* all belong to the *Mycobacterium fortuitum complex*. A study done by Velayati et al. [42] also found *Mycobacterium fortuitum* to be the most common species in water. Immune compromised individuals are more likely to be infected with *Mycobacterium fortuitum* from water sources. Infections caused by this organism include skin and soft tissue infections, lymphadenitis, pulmonary infections and catheter related infections [11].

*Mycobacterium senegalense* and *Mycobacterium peregrinum* have also been isolated in water elsewhere [20, 37]. They are potentially pathogenic NTM species and have been implicated to cause disease both in immune competent and immune compromised persons [39]. The isolation of *Mycobacterium senegalense* in water sources of Namwala district is another key finding in our study. *Mycobacterium senegalense* is a rapidly growing mycobacterium species that has been reported to cause bovine farcy among cattle in east Africa and bovine farcy has been known to cause huge economic loss [14]. Therefore, isolation of this NTM species in water in the present study poses a risk to cattle in Namwala district because this is a pastoral area [21]. Further, all *Mycobacterium senegalense* species isolated in this study were from Maala and this area has the largest number of cattle.

## Conclusion

This study has shown the isolation of NTM species from humans and water with *Mycobacterium arupense and Mycobacterium gordonae* being the most prevalent. However, there is no evidence of human to human acquisition but rather a common environmental source, water. Further, the wide presence of NTM in the water sources of Namwala could pose a risk especially for the immune compromised individuals. Therefore, prevention of public health risk of NTM should take into account water treatment before it’s accessed for domestic consumption.

## Abbreviations

AFB: Acid-Fast Bacilli
AIDS: Acquired Immunodeficiency Syndrome
CI: Confidence Interval
CPC: Cetylpyridinium Chloride
DNA: Deoxyribonucleic Acid
HIV: Human Immunodeficiency Virus
LJ: Löwenstein-Jensen
MTC: Mycobacterium tuberculosis complex
NCBI: National Centre for Biotechnology Information
NTM: Non-tuberculous Mycobacteria
PCR: Polymerase Chain reaction
TB: Tuberculosis
WHO: World Health Organization
ZN: Ziehl Neelsen
